# Enhanced comfort and biomechanical performance of ejection seat cushions via optimal double-layer foam design

**DOI:** 10.3389/fbioe.2026.1748875

**Published:** 2026-03-25

**Authors:** Yixuan Guan, Yu Duan, Pengyan Zhou, Yongqi Xie, Qiuyu Liu, Jiayi Bao

**Affiliations:** 1 Hangzhou International Innovation Institute, Beihang University, Hangzhou, China; 2 Air Force Characteristic Medical Center, Beijing, China; 3 School of Aeronautic Science and Engineering, Beihang University, Beijing, China

**Keywords:** comfort evaluation, double-layer cushion, ejection seat cushion, mechanical characterization, pressure distribution

## Abstract

**Introduction:**

Owing to inherent structural limitations of conventional single-layer ejection seat cushions, particularly their limited capacity for pressure redistribution, prolonged missions may be associated with inadequate comfort and impaired circulatory function. To address this issue, this study proposed a double-layer cushion design approach and compared three thickness ratios (1:1, 1:2, 2:1) of fast-recovery (N1) and slow-recovery (N3) foam with a single-layer N3 cushion.

**Methods:**

Two experiments, a sitting comfort test and a material compression test, were conducted to evaluate comfort and mechanical performance. The comfort test (22 participants) collected subjective ratings and interface pressure metrics, while the compression test quantified deformation and load-bearing behavior, allowing comparison of comfort and support among the different cushion structures.

**Results:**

Results showed that as N3 thickness increased, maximum pressure (*P_m_*) and average pressure (*P_v_*) decreased while contact area (*A_c_*), SPD%, and SAG factor rose; at the N1/N3 ratio of 1:2, these objective indicators reached optimal values, consistent with the highest subjective comfort ratings. In addition, the mechanical metrics matched those of the original cushion.

**Discussion:**

Overall, the double-layer design achieves an optimal trade-off between comfort and support, with the N1/N3=1:2 prototype exhibiting the superior performance. These findings provide a practical optimization strategy for developing high-performance seating systems.

## 1 Introduction

The ejection seat is a critical life-support system in combat aircraft, required to maintain pilot endurance during prolonged missions while simultaneously ensuring effective impact protection during emergency ejection (Perry et al., 2002). In practice, cushion design must reconcile two competing demands: reducing fatigue through effective pressure redistribution and maintaining sufficient structural stiffness to preserve ejection performance. However, current designs tend to prioritize impact safety over fatigue mitigation (Rahman et al., 2025), which becomes increasingly problematic as modern combat missions extend in duration and operational intensity. Addressing this balance between fatigue reduction and ejection reliability is therefore essential for improving both mission endurance and pilot survivability (Truszczyńska et al., 2014).

Among all ejection seat components, the cushion plays a central role in comfort, as it interfaces directly with the pilot’s body to distribute pressure and support blood circulation (Pellettiere et al., 2006). Its performance is heavily influenced by material selection and structural configuration (Moon et al., 2020; Akçay and Şefkat, 2023; Shi and Ma, 2024). Slow-recovery foams, originally developed for NASA’s Apollo Command Module (Yost and Oates, 1968; Luo et al., 2017), are widely used due to their viscoelasticity and open-cell structure, enabling effective pressure redistribution during extended sitting. This adaptive response contributes to localized pressure moderation and perceived comfort. In addition, the temperature-sensitive viscoelastic behavior of polyurethane foam promotes adaptive softening and improved pressure redistribution under body heat. Nevertheless, its mechanical stability and energy absorption are strongly influenced by temperature and moisture, which may accelerate aging and compromise long-term durability (Scarfato et al., 2017; Morrison et al., 2024). Furthermore, excessively compliant foam materials may increase lumbar load under vertical impact conditio ns, indicating that cushioning comfort must be balanced against impact protection performance (Adams and Safai, 2003; Wang and Dal Nevo, 2018).

In contrast, fast-recovery foams offer higher mechanical resilience but often compromise pressure uniformity and user comfort (Bao et al., 2021). Increasing overall cushion thickness to compensate for support loss is constrained by ejection seat standards. For example, thickness beneath the ischial tuberosity is typically limited to 15 mm to prevent excessive lumbar loading (Beheshti and Lankarani, 2006). Given these limitations, a hybrid cushion structure combining slow-recovery foam and fast-recovery foam presents a promising solution.

This layered concept resembles the design of composite sandwich structures, where the face sheet (analogous to contact layer) provides rigidity and the core absorbs energy (Ding et al., 2013). Functionally, the ejection seat cushion can be divided into a contact layer and a support layer (Dénes et al., 2020), which prioritizes comfort and shock attenuation, and a support layer, which maintains structural stability and prevents bottoming out. Slow-recovery foams are suited for the contact layer due to their viscoelastic behavior and high conformity, which allow effective pressure redistribution and reduction of localized stress concentrations. In contrast, fast-recovery foams exhibit higher resilience and stiffness, enabling rapid load response and improved structural support, making them more suitable for the support layer (Bao et al., 2021). This functional separation aligns well with the confined geometry and performance demands of ejection seat systems.

Several studies have examined layered cushion designs in aviation contexts from both comfort and impact perspectives. Jackson et al. (2009) used pressure-mapping techniques in a glider simulator and reported that layered viscoelastic foam structures improved long-duration sitting comfort. Perry et al. (2002), Perry (2007) evaluated seat cushions under vertical impact conditions using instrumented manikins and demonstrated that foam material properties significantly influenced lumbar load response and estimated spinal injury risk. However, these studies focused primarily on material selection and qualitative configuration comparisons, without systematically investigating the effect of layer thickness ratios on both comfort and mechanical performance.

To address this structural research gap, a reliable and multidimensional evaluation framework is required, since quantifying thickness-related effects depends on consistent and scientifically grounded assessment methods. Seating comfort is commonly evaluated through both subjective and objective approaches. Subjective evaluations often rely on local or multidimensional comfort scales (Zhang et al., 1996; Stubbs et al., 2005; Cascioli et al., 2011). Objective assessments include posture analysis (Roynarin et al., 2024), physiological signals (Dedering et al., 2002), and sitting pressure distribution (Li J. et al., 2023), which has shown the highest correlation with perceived comfort (Xu and Xia, 1997; De Looze et al., 2003). Zemp et al. (2016) and Liu et al. (2018) identified four core indicators: $P_{m}$ , $P_{v}$ , $A_{c}$ , and SPD%, widely adopted in comfort studies (Jackson et al., 2009; Smardzewski et al., 2014; Guo et al., 2016; Jiang et al., 2019; Li et al., 2020). Support performance is typically evaluated through material mechanics. Indentation force deflection (IFD) characterizes load-bearing capacity (Moon et al., 2020), while fixed-stress deformation and strain curves reflect structural stability under compression (Montalti et al., 2025).

Most previous studies have examined comfort and impact performance separately (De Looze et al., 2003; Moon et al., 2020). However, the combined influence of structural parameters such as layer thickness ratios on both ergonomic and mechanical dimensions has not been systematically quantified under aerospace-specific constraints. To address this gap, the present study proposes and evaluates a double-layer cushion integrating fast- and slow-recovery foams within the dimensional and mechanical constraints of ejection seats. Three N1/N3 thickness ratios (1:1, 1:2, 2:1) were examined using a dual-assessment framework that combines a Sitting Comfort Test and a Material Compression Test to jointly quantify ergonomic and mechanical performance.

## 2 Materials and methods

### 2.1 Double-layer cushion samples

The ejection seat cushions in this study were manufactured to match the shape and size of the Chinese fourth-generation ejection seat. Based on research from Bao et al. (2021), the slow-recovery foam N3 (model LMLR@25136), produced by a chemical research institute in Luoyang, exhibits viscoelastic conformity and effective pressure redistribution characteristics, making it suitable for use as the contact layer. In contrast, the fast-recovery foam N1 (model LMHR@25290) demonstrates higher resilience and stiffness, providing improved load-bearing capacity and structural support, which are desirable attributes for the support layer. The material properties are summarized in Table 1. Among these properties, the 25% IFD represents the force required to compress the cushion to 25% of its original thickness.

**TABLE 1:** N1 and N3 foam parameters.

Foam parameters		N1	N3
Density (kg/m^3^)		72	70
Tensile strength (Kpa)		164	168
Elongation at break (%)		96	137
Tear strength (N/m)		286	404
IFD(N)	**25%**	290	136
	**50%**	458	157
	**65%**	860	280
	**75%**	1,670	560
	**85%**	6,730	2,230

Bold represents the IFD, from 25% to 85%.

Four types of seat cushions were tested in this study: three double-layer configurations (A, B, and C) and one single-material control (O).

All double-layer cushions had a total thickness of 30 mm, composed of a fast-recovery foam (N1) as the support layer and a slow-recovery foam (N3) as the contact layer. The layer thickness configurations of the four groups are summarized in Table 2.

**TABLE 2:** Layer thickness configurations of the four cushion groups.

Group	Support layer (N1)	Contact layer (N3)	Configuration description
A	20 mm	10 mm	Thicker support layer
B	15 mm	15 mm	Equal thickness layers
C	10 mm	20 mm	Thicker contact layer
O	-	30 mm	Single-layer N3

The cutting-and-bonding method was adopted over injection molding to maintain material consistency and structural precision. This approach was necessary because slow-recovery foams are susceptible to molding conditions that could alter their internal structure (Yang and Pitchumani, 2003).

### 2.2 Sitting comfort experiment

#### 2.2.1 Participants

Twenty-two healthy male postgraduate students from Beihang University (age: 21–26 years) participated in the experiment. The ejection seat evaluated in this study is designed according to the anthropometric standards of Chinese male pilots; therefore, only male participants were recruited to ensure compatibility between body dimensions and seat geometry. Their mean weight was 64.81 ± 5.50 kg, height 172.55 ± 4.52 cm, and BMI 21.85 ± 2.01 kg/m^2^. Participants with musculoskeletal disorders, such as scoliosis or herniated discs, were excluded from the study. Participants were advised to avoid strenuous physical activity for 24 h prior to testing to minimize potential muscle fatigue. On the day of testing, participants were required to wear soft, lightweight, and close-fitting clothing to minimize the influence of garment thickness and fabric stiffness on pressure distribution measurements and to ensure uniform testing conditions. The experimental protocol was approved by the Ethics Committee of Beihang University, and all procedures were conducted in accordance with relevant guidelines and regulations. Informed consent was obtained from all participants prior to the experiment.

#### 2.2.2 Subjective comfort evaluation

Existing subjective comfort assessment methods can be broadly categorized into general, local, and multidimensional rating scales. General scales (Li et al., 2020) treat comfort and discomfort as opposite ends of a single continuum but lack sensitivity to subtle differences between similar seat configurations. Local scales assess comfort in specific body regions (Stubbs et al., 2005; Cascioli et al., 2011); however, since areas like the head, arms, and back do not interact directly with the seat cushion, such approaches offer limited relevance for seat padding evaluation. In this study, the cushion structures are largely similar, making both general and local scales unsuitable for differentiating among samples.

Instead, a multidimensional comfort rating scale was adopted, as it allows for a more comprehensive evaluation across various perceptual dimensions. This type of scale treats comfort and discomfort as independent constructs and captures nuanced fatigue-related sensations such as soreness, numbness, and stiffness (Zhang et al., 1996; Liu et al., 2018). While the original scale was developed for long-duration seating assessments, the current study focuses on short-term sitting. Therefore, a customized version of the multidimensional scale was employed to enhance sensitivity to subtle differences among cushion configurations and to minimize individual subjective bias.

The analytic hierarchy process (AHP) was applied to determine the weighting of each descriptor in the subjective evaluation scale (Bao et al., 2021), as summarized in Supplementary Appendix A. Comfort and discomfort were quantified separately, with positive scores representing comfort and negative scores representing discomfort. Given that all cushion samples were composed of similar materials and differed only in structural configuration, variations in subjective comfort perception were subtle. To supplement the evaluation, a comfort comparison questionnaire based on a customized multidimensional scale was incorporated (Ebe and Griffin, 2001), as shown in Supplementary Appendix B.

#### 2.2.3 Pressure distribution measurement

Sitting pressure distribution refers to the pattern of interface pressure between the human body and the seat surface during sitting. In this study, pressure data were collected using the CONFORMat thin-profile pressure mapping system (Tekscan, Boston, United States). Data acquisition and analysis were performed with CONFORMat Research 7.20 software.

#### 2.2.4 Experimental procedures

To eliminate order effects, the sequence of cushion testing was randomized across participants. Prior to the sitting pressure test, each subject underwent a calibration procedure using the CONFORMat Research 7.20. The pressure pad was placed on a hard, flat surface, and the participant sat gently with both feet off the ground to ensure that only the buttocks and thighs were in contact. The subject’s body weight was then entered into the software, and calibration was completed within 60 s.

At the beginning of each trial, the test cushion was placed on the ejection seat. The participant slowly sits down, leans back naturally, and places his feet on the rudder in the posture of flying. Adjust the horizontal distance of the seat so that the rudder is in a comfortable position and the feet can be freely operated. The thighs made contact with the front edge of the cushion without exerting pressure.

Participants were instructed to assess the cushion’s comfort using the provided subjective scales immediately following 60 min of continuous sitting. Subsequently, the pressure mat was laid flat over the same cushion, and the subject resumed the same posture with arms resting naturally on the thighs. Pressure distribution data were recorded for approximately 5 min at a sampling rate of 8 Hz, yielding 200 frames of data saved in video format.

After each session, participants rested for 5 min before proceeding to the next cushion. Upon completing all tests, they were asked to re-experience all cushions freely to enable relative comparison. Final subjective scores were then refined to minimize bias due to the initial lack of direct contrast.

### 2.3 Material Mechanics Compression Test

#### 2.3.1 Materials

The evaluation of seat cushion support performance was based on measurements of IFD and fixed-stress deformation. The universal testing system (MTS, United States) was used for all experiments, with an indenter accuracy of ±1 N.

#### 2.3.2 Procedures

The mechanical tests were conducted in accordance with GB/T 10807–2006 Flexible Cellular Polymeric Materials—Determination of Hardness (Indentation Technique) (Zeng and Shi, 2006). To reflect in-use conditions, all measurements were performed directly on full-size seat cushions rather than on standardized specimens. Each cushion was secured using a fixed support plate, and the indentation force required to achieve specified deformation levels was recorded as IFD.

Both IFD and strain curves under fixed stress were obtained for all four cushion configurations. IFD was used to evaluate the overall support performance, while the strain curves reflected the deformation response of the cushion under sustained loading.

To simulate the pressure exerted at the ischial tuberosity during sitting, the fixed load was determined based on the maximum peak interface pressure measured in the Sitting Comfort Experiment (1.88 N/cm^2^). To replicate this upper-bound loading condition in the material compression test, the pressure value was conservatively rounded to 2.0 N/cm^2^ for stable mechanical control.

A circular indenter with a diameter of 150 mm (radius = 7.5 cm) was used. The equivalent load was calculated according to Equations 1:

F=P×A (1)

Where $A=\pi r^{2}=176.71\;{cm}^{2}$

Therefore, the applied load was 353.25 N.

After completing the IFD measurement and allowing the cushion to fully recover, each sample was compressed at a rate of 100 mm/min until the target load was reached. The strain response was monitored continuously under the constant load, and measurements were recorded once the curve reached a plateau, indicating stabilized deformation.

Based on the strain curves under fixed-stress conditions, the following parameters were extracted:

- Initial thickness: the cushion thickness measured in its natural, unloaded state.
- Stressed thickness: the thickness recorded immediately after applying a fixed load, simulating the compressive state beneath the ischial tuberosities during sitting.
- Stabilized thickness: the thickness value after deformation reaches a steady state.
- Sagging thickness: the difference between stressed thickness and stabilized thickness, indicating the extent of post-load sinking.
- Sinkage rate: the ratio of sagging thickness to stressed thickness, reflecting the relative degree of cushion deformation under load.
- Stabilization time: the time interval required for the cushion to reach a stable thickness after loading.

### 2.4 Data processing

#### 2.4.1 Subjective rating analysis

Statistical analysis of the subjective comfort questionnaire was performed using SPSS 17.0. Outliers were first identified and excluded based on the Z-score method. The mean value of each sub-item was then calculated and multiplied by its corresponding priority scale. The weighted scores were subsequently summed to derive the overall subjective comfort score.

For the comfort comparison questionnaire, outliers were also removed using the Z-score method, followed by the calculation of the mean score for each cushion.

All results from the multidimensional comfort rating scale and the comfort comparison questionnaire were normalized prior to statistical analysis. One-way repeated-measures ANOVA was performed after confirming that the data satisfied the assumption of normality.

#### 2.4.2 Pressure distribution data processing

Four commonly used pressure distribution metrics were selected for analysis: $P_{m}$ , $P_{v}$ , $A_{c}$ , SPD%.

$P_{m}$ refers to the highest pressure value recorded among all sensor measurement points during sitting, as defined in Equation 2.

$$P_{m}=\max\left(P_{1},P_{2},\ldots ,P_{n}\right)$$ (2)

n denotes the number of measurement points.

$P_{v}$ refers to the arithmetic average of all pressure measurement points, which is calculated by Equation 3.

Pv=1n∑i=1nPi (3)

n denotes the number of non-zero pressure measurement points.

$A_{c}$ refers to the total surface area over which the body interfaces with the seat cushion during sitting.

SPD% is a pressure gradient parameter proposed by Ahmadian et al. (2002) to quantify the uniformity of pressure distribution across the seat surface. It is computationally efficient and has demonstrated good effectiveness in comfort evaluation (Hu et al., 2019; Campos and Xi, 2020; Tang et al., 2021). The calculation formula is given in Equation 4 as follows.

SPD%=∑i=1n pi−pν24npν2×100 (4)

$P_{i}$ represents the pressure value of the i -th cell, $P_{v}$ denotes the average pressure, and n signifies the total number of pressure points with non-zero values. The SPD% reflects the overall uniformity of pressure distribution on the cushion surface. Lower SPD% values correspond to more even pressure distribution, which is generally associated with improved seating comfort.

Initially, the CONFORMat Research 7.20 automatically pre-processed the video data by removing unstable frames to eliminate artifacts caused by minor subject movements at the beginning and end of each test session. Additionally, the sampling region was restricted to exclude data from deformed edge zones of the pressure mat, which may introduce inaccuracies. The pre-processed data were exported as a dataset containing approximately 130 frames, each represented by a 32 × 32 matrix of sensor values.

The $P_{m}$ , $P_{v}$ and $A_{c}$ were recorded using the software. SPD% was subsequently calculated from the dataset using MATLAB R2019a (MathWorks, United States).

All pressure metrics were normalized relative to the experimental groups. Outliers were removed using the Z-score method, and mean values were computed using the control group as the reference baseline. After verifying the assumption of normality, one-way repeated-measures ANOVA was conducted for statistical comparison.

#### 2.4.3 Data processing for compression experiments in mechanics of materials

This paper employed IFD to characterize the hardness of foam materials (Moon et al., 2020). IFD refers to the compressive stress required to compress the cushion to a fixed percentage of deformation. Lower indentation hardness at small deformations indicates greater suppleness, while higher indentation hardness at larger deformations suggests better support. IFD was measured using the mechanical testing machine, which recorded the force required to compress the cushion to various levels of deformation. This data was subsequently used to calculate the comfort factor SAG. The SAG factor was first proposed by Wolfe (Wolfe, 1982) to evaluate the comfort performance of cushions and is calculated according to Equation 5.

SAG=65% IFD25% IFD (5)

## 3 Results

### 3.1 Sitting comfort experiment results

This section summarizes the results of the sitting comfort experiment, which integrated subjective ratings and objective pressure distribution metrics to comprehensively evaluate comfort performance across all cushion configurations, as shown in Table 3.

**TABLE 3:** Summary of the comfort test results of the double-layer structure cushion samples.

Evaluation indicators	Cushion number				F	P
	O	A	B	C		
$P_{m}$	1.000	1.270	1.149	1.005	F (2.416, 50.740) = 10.517	<0.001
$P_{v}$	1.000	1.066	1.027	1.001	F (4, 84) = 5.602	<0.001
$A_{c}$	1.000	0.948	0.962	0.979	F (2.372, 49.821) = 7.489	0.001
*SPD%*	1.000	1.327	1.172	1.072	F (2.785, 58.487) = 22.642	<0.001
Subjective score	1.000	0.917	0.949	1.060	F (2.705, 56.803) = 1.381	0.259
Comparison score	0.000	0.591	0.909	1.091	F (4, 84) = 4.705	0.002

**FIGURE 1:**
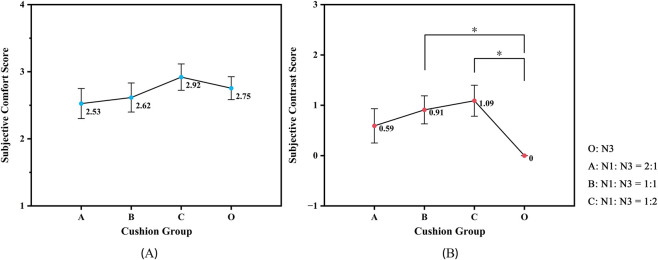
Subjective comfort evaluation of different cushion configurations. **(A)** Subjective comfort scores; **(B)** Comparative comfort scores (*, p < 0.05). Error bars represent standard error (SE) of the mean (n = 22).

Specifically, subjective comfort scores exhibited an upward trend with increasing thickness of the slow-recovery foam layer (Figure 1A); however, no statistically significant differences were observed among the cushion configurations.

In contrast, the comfort comparison scores (Figure 1B) demonstrated statistically significant differences among the cushion configurations. Notably, both Group B (p = 0.037) and Group C (p = 0.019) outperformed the control group, with Group C achieving the highest ratings in both comparative and overall subjective assessments.

**FIGURE 2:**
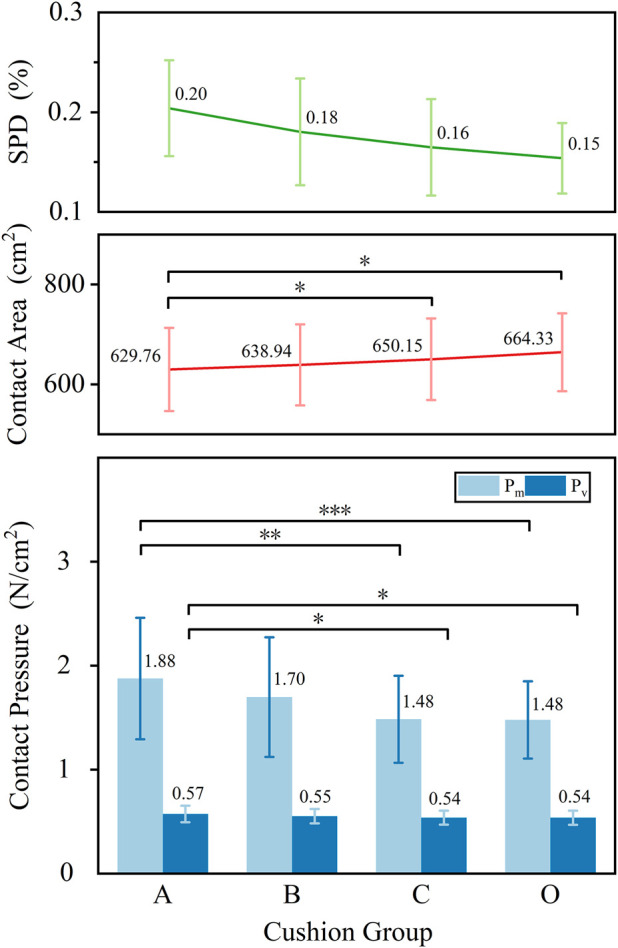
Objective sitting pressure distribution indicators (*, p < 0.05; **, p < 0.01; ***, p < 0.001).

For all objective pressure distribution indicators ( $P_{m}$ , $P_{v}$ , $A_{c}$ , and SPD%), statistically significant differences were observed among groups (all p < 0.01), as shown in Figure 2. A decreasing trend was observed in both $P_{m}$ and $P_{v}$ with increasing slow-recovery foam thickness. The control group (O) exhibited the lowest values, while the experimental groups (A to C) showed a gradual decline, with Group C approaching the control group in both metrics. Group A exhibited significantly higher $P_{m}$ and $P_{v}$ values compared to both Group O ( $P_{m}$ : p < 0.001; $P_{v}$ : p = 0.024) and Group C ( $P_{m}$ : p = 0.002; $P_{v}$ : p = 0.045).

SPD% values also showed a decreasing trend across experimental groups, with Group O presenting the lowest SPD%, and values in Groups A, B, and C gradually decreasing with increasing slow-recovery foam thickness.

The $A_{c}$ exhibited an increasing trend across Groups A to C, with the largest value observed in the control group (O). Group A exhibited a significantly smaller contact area than both Group O (p = 0.018) and Group C (p = 0.025).

**FIGURE 3:**
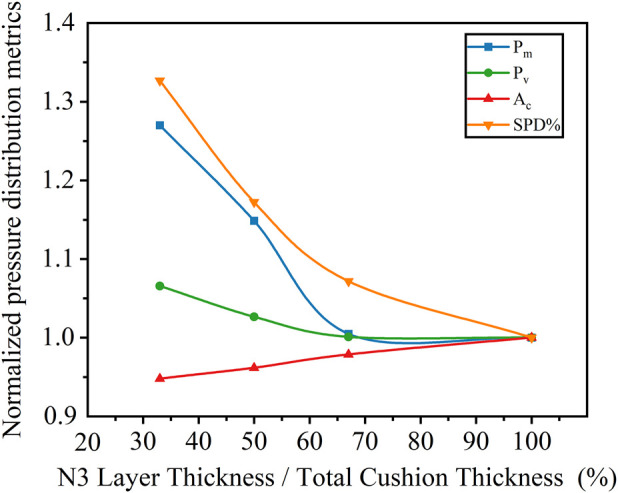
Normalized trends of sitting pressure indicators across different N1/N3 thickness ratios.

Normalized curves of the pressure-related indicators were plotted in Figure 3. Ac exhibited an approximately linear increase, whereas $P_{m}$ , $P_{v}$ and SPD% showed nonlinear decreasing trends that plateaued with increasing thickness. Both $P_{m}$ and $P_{v}$ stabilized when the N1/N3 thickness ratio was 2:1, suggesting this as the optimal configuration for balancing comfort and support.

### 3.2 Materials mechanics compression test results

The results of the Material Mechanics Compression Test are presented in Table 4. After incorporating a support layer, the support force at 65% IFD for all three experimental cushions exceeded that of the control group (O). Moreover, the overall support performance increased proportionally with the thickness of the support layer.

**TABLE 4:** Indentation test results of the double-layer seat cushion sample.

Indentationtestparameters	​	A	B	C	O
IFD(N)	25%	251.9	181.2	131.7	136.1
	50%	399.9	354.3	257.1	157.8
	65%	587.0	493.7	390.0	280.4
	75%	1,031.3	803.9	705.9	560.9
	85%	2,831.5	2016.0	1,693.4	2,230.6
SAG		2.3	2.7	3.0	2.1

To further explore comfort-related mechanical characteristics, the SAG factor was calculated for each cushion. All experimental groups exhibited higher SAG values than the control group, with Group C achieving the highest value, indicating improved cushioning characteristics.

Strain behavior under fixed-load compression was subsequently analyzed to provide additional insight into the structural support characteristics of each cushion configuration. The corresponding results are presented in Table 5.

**TABLE 5:** Fixed stress sag results summary.

Fixed stress sag parameters	A	B	C	O	Original
Initial thickness (mm)	29.68	29.67	30.45	30.35	32.98
Stressed thickness (mm)	16.62	14.48	13.12	12.19	13.10
Stabilizing thickness (mm)	14.21	11.43	9.84	8.78	9.46
Sagging thickness (mm)	2.41	3.05	3.28	3.41	3.64
Sinkage rate	0.15	0.21	0.25	0.28	0.28
Stabilization time(s)	472.76	424.58	356.03	381.01	311.86

The initial thickness of the experimental cushions was slightly lower than that of the original cushion and comparable to Group O. Under load, the stressed thickness values for Groups A, B, and C were higher than that of Group O and close to or exceeding the original. The stabilizing thickness in all experimental groups was greater than in Group O and the original cushion, with Group A reaching 14.21 mm, B at 11.43 mm, and C at 9.84 mm. Sagging thickness was lower in Group A and B than in Group O and the original cushion, while Group C showed similar values to O. Sinkage rates followed a gradual increase from Group A to C, remaining lower than O and the original. Stabilization time was longer in Groups A and B compared to both Group O and the original cushion, while Group C fell between O and B.

## 4 Discussion

This study employed two complementary experiments to systematically evaluate the ergonomic and mechanical performance of double-layer cushion prototypes under aerospace-specific constraints. Comfort was assessed using both subjective and objective indicators, including $P_{m}$ , $P_{v}$ , $A_{c}$ , SPD%, and the SAG factor. Support performance was characterized through strain-curve-derived metrics under fixed-stress conditions, such as stressed thickness, stabilizing thickness, and stabilization time.

The integration of these dual assessments enabled a comprehensive examination of how different material combinations and thickness ratios affect both perceived comfort and mechanical resilience. Notably, the comfort-related outcomes revealed several important trends regarding the influence of slow-recovery foam thickness. Subjective comfort scores tended to increase with greater slow-recovery foam thickness; however, no statistically significant differences were observed across the cushion configurations. This lack of significance is likely attributable to the uniformity of the contact layer material across all groups and the limited structural variation in the prototypes. Additionally, the short-duration sitting protocol may have reduced perceptible differences, which are more likely to emerge during prolonged use, when mechanical support plays a greater role in overall comfort perception. The weighted nature of the multidimensional comfort scale may have further limited its sensitivity to subtle structural distinctions. In contrast, the comparative comfort evaluation revealed statistically significant distinctions between groups. Group B and Group C demonstrated notably higher comparative comfort scores than the control, with Group C exhibiting the highest overall ratings. This suggests that the comparative evaluation method may be more sensitive than absolute scales in detecting nuanced differences among structurally similar cushions. The superior performance of Group C highlights the benefit of increasing the slow-recovery foam thickness in enhancing subjective comfort, suggesting that relative comparison frameworks can amplify user-perceived differences under controlled conditions.

This trend in subjective comfort ratings is further corroborated by the objective sitting pressure distribution data, which demonstrated consistent improvements with increasing slow-recovery foam thickness. Specifically, $P_{m}$ , $P_{v}$ , and SPD% showed negative correlation with subjective comfort scores, while $A_{c}$ was positively correlated (Fang et al., 2016; Li X. et al., 2023).

A clear downward trend in $P_{m}$ and $P_{v}$ was observed across the experimental groups, indicating reduced localized pressure and a softer seating interface (Smulders et al., 2016; Silva et al., 2024). Similarly, SPD% gradually decreased with increasing slow-recovery foam thickness, reflecting a more uniform pressure distribution, which is a key factor in long-term sitting comfort (Smardzewski et al., 2014; Hu et al., 2019). In contrast, Ac progressively increased, suggesting that thicker slow-recovery layers enable broader body–cushion contact. This contributes to better pressure dispersion and minimizes localized stress concentrations. These findings are consistent with previous research by Dangal et al. (2021), Silva et al. (2024), which reported a positive association between larger contact areas and improved perceived comfort.

These objective results are further reinforced by the SAG factor, a derived metric from indentation force deflection, which reflects the cushion’s ability to combine softness with mechanical resilience. Group C exhibited the highest SAG factor among all configurations, indicating superior comfort through enhanced thickness of the contact layer. As a biomechanical proxy for perceived softness, the SAG factor has demonstrated strong predictive power across domains such as bedding, office, and automotive seating (Cunningham et al., 1994; Ebe and Griffin, 2001; Wang et al., 2013; Shen et al., 2014; Moon et al., 2020), and proves equally applicable to aerospace contexts in this study.

Together, the convergence of subjective ratings, pressure distribution indices, and the SAG factor consistently identifies the 2:1 configuration (Group C) as the most ergonomically favorable (Smardzewski et al., 2014; Fang et al., 2016; Smulders et al., 2016; Dangal et al., 2021). Beyond this thickness ratio, further increases in the slow-recovery layer provide minimal additional gains in comfort. This observation aligns with findings by Mohanty and Mahapatra (2014), which indicated that comfort improves with cushion thickness up to approximately 6–8 cm, beyond which no significant enhancement is observed.

Beyond its impact on perceived comfort, cushion configuration also plays a critical role in determining mechanical support characteristics, particularly under sustained or dynamic loading. While increased slow-recovery foam thickness enhances pressure dispersion and user comfort, it may also affect structural behavior under load. To ensure that these ergonomic improvements do not compromise support integrity, this study further analyzed strain-curve-derived mechanical parameters under fixed-stress conditions.

The results of the material compression tests underscore the importance of structural configuration in enhancing seat cushion support. The increased support force observed at 65% IFD across all experimental groups indicates that the incorporation of a fast-recovery foam support layer significantly strengthens load-bearing capacity. This is particularly notable in Group C, which exhibited the highest IFD values, confirming that greater support layer thickness directly translates to improved mechanical resilience, a finding consistent with the observations of Moon et al. (2020) in multilayer cushion systems.

Beyond force-based assessments, deformation behavior under fixed-load conditions offers additional insights into support effectiveness. The sinkage rate increased with greater contact layer thickness, reflecting the greater compliance of the slow-recovery foam under localized load. However, Cushion A’s low sinkage rate suggests enhanced resistance to shape collapse, which may be advantageous for maintaining postural stability during high-G acceleration. Conversely, the thicker contact layer configurations, while more compliant, may offer better long-term pressure dispersion at the expense of slightly increased deformation.

The stabilization time, defined as the time required for the cushion to reach equilibrium under sustained load, was shortest in Group C. This rapid transition to mechanical stability suggests better dynamic response, which is desirable in aerospace applications subject to variable forces. Moreover, Group C also achieved the highest stabilized thickness, implying greater rebound capacity and energy absorption over prolonged use. A thicker residual profile after loading ensures adequate support for the lumbar spine and pelvis, particularly important during ejection or extended missions.

Taken together, these findings demonstrate that the double-layer cushion configuration, especially with a 2:1 fast-to-slow foam thickness ratio, maintains or even improves structural support compared to conventional single-layer designs. By optimizing both mechanical stiffness and strain recovery characteristics, this design offers a compelling solution for applications requiring both high comfort and robust support performance.

To fully realize the benefits of the double-layer cushion structure, the selection of an appropriate cushion cover material is equally critical. The cushion cover should not only have good ventilation and heat dissipation function, but also have good ductility to ensure the effectiveness of slow-recovery materials. In consideration of the material properties and the feasibility of production, a soft and breathable high elastic synthetic fiber was selected for the cushion cover. Silva et al. (2024) have confirmed through a thermographic assessment that fabric covers can produce lower temperatures at passenger interface contacts, resulting in improved comfort.

Although the present Sitting Comfort Experiment employed a standardized 60-min protocol to ensure controlled evaluation conditions, operational flight scenarios may involve substantially longer seated durations. The long-term stability of pressure redistribution and structural support performance under extended exposure therefore warrants further investigation. Future work will incorporate extended-duration sitting assessments to validate the temporal robustness of the proposed cushion configuration.

Beyond time-dependent validation, further biomechanical investigation is also necessary to examine load transmission mechanisms under extreme operational conditions. Future studies will incorporate coupled human–cushion finite element modeling to simulate spinal stress and load transfer under ejection or high-acceleration scenarios, thereby further verifying the protective performance of the proposed structure. Machine learning–assisted approaches may additionally be employed to accelerate simulation-based prediction and support rapid assessment of injury risk and personalized structural optimization (Zhang et al., 2025).

## 5 Conclusion

This study evaluated a double-layer ejection seat cushion combining a fast-recovery support layer (N1) and a slow-recovery contact layer (N3) using an integrated ergonomic–mechanical assessment framework. Among the tested configurations, the 1:2 N1/N3 thickness ratio demonstrated the most favorable balance between pressure redistribution, perceived comfort, and structural support. The dual-indicator evaluation approach confirmed the consistency between subjective and objective measures and provided a systematic method for optimizing cushion design under aerospace constraints. Overall, the proposed double-layer structure achieves an effective balance between comfort and support and offers a practical design solution for high-performance seating systems.

However, several limitations should be acknowledged. The present study was conducted under controlled laboratory conditions and focused on short-duration sitting assessment using healthy male participants. Dynamic safety performance under ejection loading and long-term sitting comfort were beyond the scope of this phase of the study. Future work will incorporate finite element analysis and anthropomorphic dummy impact testing to further validate spinal load response under ejection conditions, as well as extended-duration sitting experiments to assess long-term comfort stability.

## Data Availability

Due to the raw data’s commercial sensitivity, it cannot be made publicly available, but de-identified data supporting key findings may be obtained from the corresponding author upon reasonable request, subject to non-disclosure agreements and for non-commercial research. Requests to access the datasets should be directed to JB, 11183@buaa.edu.cn.
