# Supplementary material for: Enhanced comfort and biomechanical performance of ejection seat cushions via optimal double-layer foam design
Source: Front Bioeng Biotechnol. 2026 Mar 25;14:1748875. doi: 10.3389/fbioe.2026.1748875 (PMC13057320; doi:10.3389/fbioe.2026.1748875)
Supplement: Supplementary file 1 [file Supplementaryfile1.docx]

# Appendices

Appendix A. Multidimensional Comfort Rating Scale

|  | Descriptors | Priority scales | Scores |
| --- | --- | --- | --- |
| Comfort | I feel relaxed | 0.04 |  |
|  | I feel spirits soared | 0.04 |  |
|  | I feel restful | 0.04 |  |
|  | I feel softer | 0.08 |  |
|  | I feel supported enough | 0.08 |  |
|  | I feel refreshed | 0.08 |  |
|  | I feel comfortable | 0.15 |  |
| Discomfort | I have sore muscles | -0.04 |  |
|  | I have heavy legs | -0.04 |  |
|  | I feel stiff | -0.04 |  |
|  | I feel tired | -0.04 |  |
|  | I have swollen ankles | -0.04 |  |
|  | I feel numb | -0.04 |  |
|  | I feel the circulation to legs cut off | -0.04 |  |
|  | I feel cramped | -0.04 |  |
|  | I feel restless | -0.04 |  |
|  | I feel uncomfortable | -0.13 |  |

*The rating scores ranged from 1 to 9, meaning from “not at all” to “extremely”.*

Appendix B. Comfort contrast questionnaire

| Cushion number | Scores |
| --- | --- |
| O |  |
| A |  |
| B |  |
| C |  |
| Descriptions:  +3: The cushion is very much more comfortable than the control.  +2: The cushion is definitely more comfortable than the control.  +1: The cushion is slightly more comfortable than the control.  0: The cushion is the same comfort as the control.  -1: The cushion is slightly less comfortable than the control.  -2: The cushion is definitely less comfortable than the control.  -3: The cushion is very much less comfortable than the control. | |
